# Biosurfactants as Antibiofilm Agents for Medical Devices: Mechanisms, Evidence and Integration into Infection Prevention and Control

**DOI:** 10.3390/microorganisms14040910

**Published:** 2026-04-17

**Authors:** Sunday Stephen Abi, Ibrahim M. Banat

**Affiliations:** 1 Independent Researcher, Nottingham NG7 7JG, UK; abisundaystephen@gmail.com; 2 School of Biomedical Sciences, Ulster University, Coleraine BT52 1SA, UK

**Keywords:** biofilm, biosurfactants, antimicrobials, antiadhesion, infection prevention, medical devices, healthcare

## Abstract

Biofilms rapidly form on medical devices such as urinary catheters and surgical materials. These biofilms compromise patient safety and undermine infection prevention and control (IPC). Biofilms also reduce the effectiveness of antibiotics and disinfectants. As a result, they increase healthcare-associated infections and increase costs through device failure and the need for maintenance or replacement. Researchers are increasingly exploring biosurfactants (BSs) as surface coatings and cleaning additives to prevent microbial attachment and disrupt early biofilm formation on medical devices and healthcare-related surfaces. This review examines the translational potential of biosurfactants as preventive, disruptive, and adjunctive antibiofilm agents for medical devices and healthcare-related surfaces. Literature evidence on glycolipids (rhamnolipids, sophorolipids) and lipopeptides (surfactin) from static, flow-based, and microfluidic in vitro models that used clinically relevant materials, such as silicone and polydimethylsiloxane (PDMS), were examined. In our literature search, we focused on pathogens central to IPC, such as *Staphylococcus aureus*, *Pseudomonas aeruginosa*, *Enterococcus* spp., and *Candida* spp., and it was generally noted that BSs reduced microbial adhesion and delayed early biofilm formation on medical devices and healthcare-related surfaces. Significant evidence also suggests that they partially disrupt biofilms and improve antimicrobial penetration when co-applied, mainly through membrane disruption, destabilization of extracellular substances, interfering with quorum sensing, and synergistic and/or antagonistic interactions with other molecules. Their performance varied with class, formulation, hydrodynamic conditions, and microbial composition. BSs function better as preventive and adjunctive IPC tools than stand-alone antimicrobial agents and can help to reduce biofilm formation on devices and improve surface disinfection. However, translating this promise into practice demands more robust data on long-term safety, stability, and product quality.

## 1. Introduction

Antimicrobial resistance (AMR) is a long-standing microbial challenge and an increasing threat to global public health and modern medicine [1,2]. Researchers from various fields worldwide have reported the presence of antimicrobial-resistant bacteria across diverse settings and reservoirs. Environmental sources such as underground human fecal storage cisterns [3], clinical surfaces [4], medical devices [5], and hospital and municipal wastewater systems [6] have been reported as sources of antimicrobial-resistant bacteria. A major concern is that biofilms on medical devices and clinical surfaces reduce antimicrobial penetration and promote tolerant subpopulations, making eradication difficult after infections are established [7,8]. Such factors contribute to the global AMR burden. Although antimicrobial discovery and research have traditionally focused on killing microorganisms, increasing attention is shifting toward preventive strategies that target biofilm formation to reduce infection risk and transmission [1,9,10]. The need for multidisciplinary collaboration to address AMR is increasingly recognized [11].

Biofilms are structured communities of microorganisms embedded in self-generated slimy material called extracellular polymeric substance (EPS) [12,13]. The EPS matrix is responsible for the unique ability of biofilms to resist antimicrobial activity and persist in infections [12,14]. While they have been explored and play immense roles in bioremediation, food fermentation, agriculture, wastewater treatment, and other fields [15], they pose a significant burden to modern medicine because of their ability to invade medical devices and healthcare-related surfaces [8,16]. Owing to their synergy and coordinated interactions, biofilms persist on surfaces, tolerate antimicrobial exposure, and evade host immune responses, hence creating significant challenges for healthcare professionals, patients, and service users (as is common in health and social care settings) [7,16,17]. Moreover, in vivo, biofilms are being increasingly recognized as heterogeneous communities embedded within host environments, where microbial and host components contribute to their structure and persistence [18,19]. Compared with planktonic cells, microorganisms within biofilms exhibit increased tolerance to antibiotics, disinfectants, and immune mechanisms because of restricted antimicrobial penetration, altered metabolic states, stress responses, and the presence of persister cells [7,14,20]. Since microbial proliferation starts with adhesion to hosts or surfaces, from an infection prevention and control (IPC) perspective, targeting early microbial adhesion and biofilm establishment may offer a more sustainable and preventive approach than attempting to eradicate mature biofilms [20,21,22].

Preventing infection is becoming a greater focus in IPC, especially by altering device or material surfaces to make it difficult for microorganisms to attach and proliferate, or to form biofilms. Surface alterations can reduce the effectiveness of conditioning films, disrupt biofilm structural integrity, and weaken the physical forces that help biofilms adhere to materials [23,24]. This fits well with IPC’s strategic goals of lowering risk, controlling sources of infection, and enhancing antimicrobial stewardship.

BSs are surfactants of microbial origin that can modify interfacial tension, surface hydrophobicity, and wettability [25]. Through these physicochemical effects, BSs can interfere with microbial adhesion and early biofilm formation [26]. Additionally, compared with synthetic surfactants, BSs are thermostable, less toxic, and biodegradable. Moreover, some BSs have been shown to be biocompatible [27,28,29,30].

Many BSs also exhibit biological activities, including membrane disruption, extracellular matrix destabilization, and modulation of microbial behaviors such as quorum sensing [26]. Rather than functioning primarily as alternative antimicrobial agents, BSs may serve as preventive and adjunctive tools, reducing microbial attachment and weakening early biofilms, thereby enhancing the effective penetration of antimicrobial agents. These findings position BSs as interventions that complement existing IPC strategies, rather than as replacements for conventional antimicrobial agents [31].

Glycolipids such as rhamnolipids (RLs), sophorolipids (SLs), and lipopeptides such as surfactin (SF) are typical BSs that have been investigated on healthcare-relevant materials, including silicone and PDMS. They are regularly explored as coatings, rinses, and lock or flush solutions [32,33,34].

In this review, we examine BSs as antibiofilm agents for medical devices from a translational IPC perspective. We synthesize evidence on key BS classes, outline mechanistic pathways, evaluate healthcare-relevant biofilms, and discuss practical applications on medical devices and healthcare-related surfaces. We also propose a conceptual IPC framework and emphasize that it should be integrated into existing IPC bundles. We then examine safety and governance considerations and the research priorities needed to support the responsible use and clinical translation of BSs.

We focus primarily on RLs, SLs, and SF, and briefly on *Lactobacillus*-derived BSs, because they have been most extensively evaluated in BS-biofilm healthcare models using materials such as silicone and PDMS against common healthcare pathogens such as *S. aureus*, *P. aeruginosa*, *Enterococcus* spp., and *Candida* spp. [35,36,37].

### 1.1. Biofilm Biology and Tolerance in Healthcare Settings

Biofilm development typically progresses through well-characterized stages: initial reversible attachment; irreversible aggregation of microbial cells to form a microcolony; and EPS production, maturation, and dispersal [12,13,38,39,40]. In clinical environments, these processes often occur on medical devices made from materials such as silicone, PDMS, polyurethane, and stainless steel. The EPS matrix of a biofilm, which is composed of polysaccharides, proteins, extracellular DNA (eDNA), and lipids, provides structural stability and protection against antimicrobial penetration and environmental stress [14,16,41]. Key healthcare-associated pathogens such as *S. aureus*, *P. aeruginosa*, *Enterococcus* spp., and *Candida* spp. readily form biofilms on medical devices and healthcare-related surfaces [14,20,42,43].

Biofilm tolerance differs from that of classical AMR mechanisms. This (mainly phenotypic) tolerance arises from restricted antimicrobial penetration by the EPS matrix, reduced metabolic activity of embedded cells, activation of stress responses, and the presence of dormant persister cell subpopulations rather than stable genetic resistance mechanisms [7,12]. These features allow biofilms to survive exposure to high concentrations of antibiotics, disinfectants, and host immune factors, making both infection prevention and treatment difficult in healthcare settings.

### 1.2. Medical Devices as Biofilm Reservoirs

Invasive and semi-invasive medical devices such as vascular catheters, urinary catheters, prosthetic joints, cardiac implants, endotracheal tubes, and silicone-based prostheses are widely used in clinical practice [44,45]. These medical devices are used in the diagnosis, treatment, and monitoring of patients both within and outside hospital settings. In a recent publication, the World Health Organization (WHO) stated that the global market of medical devices includes an estimated 2 million different types, grouped into more than 7000 generic device categories [46]. Many of these medical devices can become favorable surfaces for microbial colonization because of prolonged exposure to biological fluids, repeated handling, and device structure [14,16].

Biofilms are involved in a large proportion of medical device-associated infections (MDAIs). Catheter-associated urinary tract infections (CAUTIs) and central line-associated bloodstream infections (CLABSIs) are common MDAIs that contribute significantly to biofilm-related healthcare-associated infections (HCAIs) [42,47]. Once biofilms are established on medical devices, they not only impair device function but also promote persistent infections. Some of these biofilms are recalcitrant to traditional antimicrobial agents and disinfectants, necessitating the removal or replacement of medical devices [8,16]. Therefore, biofilm-associated infections increase morbidity, prolong hospital stays, increase healthcare costs, and contribute to AMR [1,16].

### 1.3. Limitations of Conventional IPC Approaches in Biofilm Control

Traditional IPC strategies rely heavily on the systemic use of antibiotics, topical antiseptics, and disinfectants [48]. While these measures are effective against planktonic microorganisms, they often fail to eradicate mature biofilms, especially within device lumens, micro-crevices, or irregular surfaces, where antimicrobial agents cannot easily penetrate, but which are potential habitats of biofilms [7,8,16]. Increasing antimicrobial or biocide concentrations may pose risks such as toxicity, material degradation, occupational exposure, and selection pressure that further promote AMR [16,48]. These limitations highlight the need for preventive approaches (as shown in Table 1) that target early stages of microbial attachment [20,21]. As summarized in Table 1, the proposed IPC framework classifies BS applications into preventive, disruptive, and adjunctive strategies. Each category is linked to specific mechanisms, use cases, safeguards, and translational readiness levels.

## 2. BSs and Their Relevance to IPC

Microorganisms such as bacteria, fungi, and yeast produce surface-active molecules as part of their lifestyles. These amphiphilic biomolecules, called BSs, are microbial secondary metabolites that can confer resistance against biofilms and AMR in humans. Some in vitro studies have demonstrated that BSs are eco-friendly, sustainable, and biocompatible [27,28,37]. All these attributes make BSs ideal for IPC in medical devices and healthcare settings. Their unique interfacial activities also make them promising candidates for preventing and disrupting biofilms on medical devices and healthcare-related surfaces. Multiple studies have shown that they are effective under diverse environmental conditions, which is a strong attribute that can be explored to achieve IPC objectives aimed at reducing HCAI [37,49]. Their amphiphilic structure enables them to interact effectively with both hydrophobic and hydrophilic interfaces [50]. This quality enables modification of surface energy, interference with microbial adhesion, and disruption of biofilm EPS. These highlighted properties position BSs as preventive and adjunctive tools, rather than direct replacements for conventional antimicrobial agents.

### 2.1. Overview of BS Production

BSs are synthesized through microbial fermentation processes, often under nutrient-limited conditions (e.g., phosphate deprivation or the presence of hydrophobic substrates), to increase secondary metabolite production in selected microorganisms [51,52]. The production pathways depend on the desired BS class and microbial source. The microbial source used and the quality of the resulting BS output strongly influence the congener composition, physicochemical properties, and translational suitability for medical device applications [50].

RLs are typically produced by bacteria in the presence of hydrophobic substrates such as vegetable oils and hydrocarbons [53,54]. In contrast, SLs are typically produced by yeast fermentation using glucose and fatty acid substrates under controlled biotechnological conditions [54,55].

SFs are produced during the stationary growth phase of *Bacillus* species in peptide- and lipid-rich media, with some anaerobic-capable strains (e.g., from oil reservoirs). BSs of a Lactobacillus source may be obtained from cell fractions or culture supernatants grown in nutrient-rich media, sometimes using low-cost substrates such as lactose or dairy byproducts [26,49,56,57]. Evidence from lactic acid bacteria grown on whey also indicates that agro-industrial dairy waste can enable cost-effective biosurfactant production, which is important for future scaling and sustainability [58].

Despite the strong potential for BSs in healthcare applications, relatively high costs are associated with the substrates required to initiate production, as well as their subsequent purification [30,59]. BS yields can vary significantly across strains, and congener heterogeneity influences surface activity and biological performance, which may lead to variability in efficacy between batches. Manufacturing provenance also has regulatory implications; for example, RLs derived from *P. aeruginosa* may raise safety concerns that require alternative producers or rigorous purification strategies because of the potential pathogenicity of the strains [60].

Researchers are exploring advances in BS process optimization, genetic engineering, and metabolic engineering to increase yield and specificity, and the use of sustainable agro-industrial waste to improve production scalability, cost-effectiveness, and regulatory compliance [61]. Comprehensive knowledge of BS production pathways is therefore critical for interpreting mechanistic differences among classes and for evaluating their translational potential within IPC frameworks [62].

These production-related factors influence the physicochemical behavior of BSs and their ethical and regulatory acceptance. The next section, therefore, provides an essential context for understanding their antibiofilm mechanisms, which will influence decision-making in the adoption of BSs for biofilm control and management in healthcare.

### 2.2. Key BSs Used Against Biofilm

BSs are broadly classified as either low-molecular-weight (LMW) or high-molecular-weight (HMW) molecules of microbial origin (e.g., bacteria, fungi, and yeast). LMW BSs have a molecular weight ranging from approximately 500 to 1500 Da [63]. BSs of LMW, such as glycolipids (e.g., SLs and RLs), and lipopeptides (e.g., SF), are relevant in biofilm control because of their strong ability to interact with and modify membranes. These factors make them relevant bioagents for preventing HCAIs and MDAIs, which are commonly caused by biofilms. However, HMW polymeric BSs (such as emulsans and liposans) and particulate BSs (such as vesicles) are not often studied for controlling biofilms on medical devices, although they could help with surface preparation and stabilization of emulsions [64].

#### 2.2.1. Glycolipids

Glycolipids are carbohydrate moieties linked to a lipid tail, of which RLs and SLs represent the most extensively investigated subclasses for medical device applications [50,65,66].

RLs are primarily produced by *P. aeruginosa* and consist of one or two rhamnose units attached to β-hydroxy fatty acids (mono- and di-rhamnolipids). RLs can alter surface hydrophobicity, reduce surface tension, and interfere with microbial adhesion. When RLs are immobilized on medical materials such as silicone or PDMS, they can reduce adhesion and early biofilm formation by modulating surface energy [67,68].

SLs are produced by *Starmerella bombicola* (formerly *Candida bombicola*). SLs are composed of sophorose disaccharides linked to hydroxy fatty acids [51]. They exist in lactonic and acidic forms, both of which demonstrate antiadhesive properties and the ability to weaken the EPS matrix. Independent studies by Díaz De Rienzo et al. [69] and Ceresa et al. [33] have shown that SLs strongly reduce microbial adhesion and promote biofilm detachment on medical devices of polymer origin, such as silicone and PDMS.

#### 2.2.2. Lipopeptides

Lipopeptides (e.g., SF) are produced by Bacillus species, predominantly by *B. subtilis* [56]. These BSs combine strong surface activity with membrane interactions, thereby inhibiting microbial adhesion and partially disrupting biofilms. Their activity is particularly relevant for fungal and mixed microbial communities, where membrane interactions and physiological sensitization contribute to preventing colonization [56,70].

#### 2.2.3. Lactobacillus-Derived BSs

BSs derived from *Lactobacillus species* (e.g., *L. rhamnosus*, *L. brevis*, *L. acidophilus*, and *L. jensenii*) often consist of glycolipid or glycolipopeptide structures. These compounds demonstrate strong antiadhesion activity on silicone, plastic, and PDMS surfaces and have been investigated against pathogens, including *S. aureus*, *Candida* spp., and other healthcare-associated microorganisms. Their probiotic origin and favorable biocompatibility profiles make them promising candidates for preventive surface conditioning within IPC strategies for preventing biofilm formation [35,71,72,73].

### 2.3. How BSs Act Against Biofilms

BSs exert antibiofilm effects through multiple mechanisms, including the modification of surface properties to reduce microbial adhesion; disruption of the EPS matrix, leading to weakened biofilm cohesion and detachment; interference with quorum sensing; and, in some cases, direct antimicrobial actions such as membrane or cell wall damage [26,74,75].

Numerous studies have shown that RLs, SLs, and SF exhibit strong effects against biofilms on medical device polymers when used as adjuncts with antibiotics or similar surfactants [76,77,78]. These effects can prevent initial biofilm formation, disperse pre-existing biofilms, and enhance the efficacy of antimicrobial agents when used in combination. These mechanisms are suitable for preventive intervention by modifying surface–microbe interactions and enhancing susceptibility to adjunct antimicrobial strategies, rather than acting as standalone bactericidal agents [79,80,81,82,83].

#### 2.3.1. Surface Energy Modulation and Antiadhesion Effects of BSs

Surface energy refers to the adhesive force or interfacial attraction that binds materials to a solid surface [84]. In the context of medical devices, it is the binding force holding a microbial cell or aggregate of microbial cells (e.g., biofilms) onto a medical device or any relevant surface within healthcare settings. In separate and independent studies, Zhao et al. [85] and Cheng et al. [86] suggested that strong relationships exist between microbial cells and surfaces, and emphasized that modifying surface energy can hinder microbial attachment. Depending on the type of material considered, a reduction in the device surface in the range of 20–40 mN/m can inhibit microbial adhesion [35,36,84,87,88,89]. Consequently, healthcare professionals may reduce the surface energy of medical devices to a range that would inhibit microbial adhesion.

BSs are sustainable tools for modifying surface physicochemical properties, such as surface energy [26]. BSs reduce surface hydrophobicity and alter interfacial energy, thereby weakening microbial attachment to typical medical device materials such as silicone and PDMS [34,67]. By shifting wettability and reducing surface susceptibility to protein adsorption, BSs can help to suppress the establishment of irreversible biofilms [27,34,67].

RLs, SLs, and SF coatings on medical devices/medical device surrogates have demonstrated strong durability and significant reductions in biofilm adhesion and biomass under both static and flow conditions. These findings highlight the importance of stable surface functionalization for translational applications in IPC [27,32,67]. Additionally, SL precoatings have been shown to reduce bacterial and fungal adhesion on silicone surfaces. This finding reinforces the role of surface energy modulation as a central preventive mechanism by BSs [33,34].

#### 2.3.2. BS Destabilization of the Biofilm EPS Matrix

BSs can interact with EPS components such as polysaccharides, eDNA, proteins, and lipids, leading to increased hydration and loosening of the EPS matrix cohesion of the biofilm, which then reduces their mechanical stability and, consequently, infection risk [67].

SLs have demonstrated the ability to weaken EPS structure and promote the detachment of established biofilms under flow conditions. Microfluidic studies involving *P. aeruginosa biofilms* have shown that EPS destabilization may result in strong detachment of microbial cells from surfaces, even when direct bactericidal activity is limited [78,90,91]. This strongly demonstrates the physicochemical disruption mechanism of BSs.

Dong et al. [92] reported an in vitro study showing that azithromycin/RLs nanoparticles effectively destabilized the *P. aeruginosa* EPS matrix by interfering with its protein and polysaccharide components. The study also demonstrated that the combined use of azithromycin and RL dispersed bacteria and prevented their attachment to host cells. Similarly, SF is a promising BS that can weaken biofilm EPS by downregulating genes such as *icaA* and *icaD*, which increase quorum sensing, EPS formation, and intercellular adhesion [93]. The findings from these studies typically support the efficacy of BSs as adjuncts to standard antimicrobials, facilitating the disruption of early biofilms and inhibiting microbial attachment.

#### 2.3.3. BS Interactions with Membranes and Physiological Sensitization of Biofilms

BSs form micelles through their amphiphilic structure. These micelles, which insert into the biofilm structure, increase permeability, dissipate the proton motive force, and cause leakage of intracellular contents [94]. Lipopeptides such as SF and lichenysin are known for their ability to physiologically sensitize biofilm cells. In medical device models, they mainly prevent colonization and adhesion rather than total eradication of established biofilms, indicating that BSs are better recognized as preventive or sensitizing agents [36,70].

#### 2.3.4. Biofilm Signaling Interference and Virulence Modulation by BSs

BSs can interfere with biofilm signaling mechanisms. For example, SF modulates the AI-2 quorum-sensing system in *S. aureus*, which leads to communication breakdown within the biofilm [93]. The alteration of QS activity significantly affects biofilm structure and metabolic activity, among others. These conditions make biofilms susceptible to antimicrobial agents. BSs’ ability to interfere with QS is not limited to SF [95]. RLs have also been shown to strongly downregulate the *agrA* and *agrC* genes in methicillin-resistant *S. aureus* (MRSA) ss [96]. In addition, BS from *L. acidophilus* reduces the expression of QS-regulated virulence factors in *P. aeruginosa*, thereby reducing the ability of the bacterium to form biofilms. Such characteristics of BSs can be harnessed to combat biofilm formation in medical devices and enhance IPC as well as antimicrobial stewardship [97].

#### 2.3.5. BS Synergy with Antibiotics, Antifungals, and Detergents

BSs frequently enhance antimicrobial penetration into biofilms through combined membrane disruption and EPS modification. As stated below, synergistic interactions have been demonstrated between SLs and detergents such as sodium dodecyl sulphate (SDS) [78].

Synergy has been reported with antibiotics such as tetracycline and cefaclor, as well as with antifungal agents when combined with lipopeptides in silicone-based models [78,98,99].

SLs have also demonstrated synergy with antibiotics. In vitro studies revealed that SLs increased bacterial membrane permeability, enhancing the activity of tetracycline against *S. aureus* and that of cefaclor against *Escherichia coli* [99]. In microfluidic studies, SLs combined with sodium SDS disrupted *P. aeruginosa* biofilms at concentrations approximately 100-fold lower than when either compound was used alone [78].

In addition, RL-functionalized PDMS coatings have been shown to reduce mono- and dual-species biofilm formation on device-relevant polymers, supporting their use as preventive surface modifications in medical devices [32].

Lipopeptide BSs also display promising synergistic interactions with antifungal agents; for example, the AC7BS lipopeptide enhanced the activity of amphotericin B and fluconazole against *C. albicans* biofilms formed on silicone surfaces, reducing the sessile minimum inhibitory concentration (SMIC) [98]. Similar adjunctive effects have also been reported against *Candida glabrata* biofilms, where Bacillus-derived lipopeptides AF4 and AF5 disrupted mature biofilms and further improved biofilm control when combined with fluconazole [100].

These findings indicate that BSs function as adjuncts with standard antimicrobial agents and work synergistically with them, inhibiting biofilm formation on medical devices. Such BS–antimicrobial synergy enhances device maintenance and IPC workflows by weakening biofilm integrity and improving antimicrobial penetration. In this way, the adjunctive role of BSs directly consolidates antimicrobial stewardship by reducing the potential doses of antimicrobials that may have been used to disrupt biofilms or prevent microbial adhesion.

#### 2.3.6. Translational Potential of BSs

BS effectiveness against biofilms in laboratory experiments may differ in clinical contexts. Hydrodynamic conditions can influence biofilm architecture and removal [101]. Moreover, compared with physical adsorption, covalent grafting improves retention and performance [102]. When implants come into contact with human body fluids, they become coated with a thin layer of proteins [103]. This can reduce antiadhesion effects unless the surface is designed to remain stable and resist fouling. In addition, different BSs work better against different types of microorganisms.

The source materials and types of microorganisms used in BS production can determine their critical micelle concentration (CMC), surface activity, and overall effectiveness [2]. Therefore, researchers should report these physicochemical parameters together with biological outcomes, which will enhance reproducibility and regulatory evaluation [60,65,104,105].

BSs that act on membranes require careful safety evaluation before medical use [106]. Some preparations show hemolytic activity at higher concentrations, but covalent grafting reduces leachable fractions and improves cytocompatibility [28,74,107,108]. In essence, these factors explain why BSs are better positioned as preventive coatings or adjuncts in IPC strategies. The next section compares BS classes from this translational perspective.

### 2.4. Comparative Translational Assessment of BS for IPC on Medical Devices

The effectiveness of BSs in the control of biofilms on medical devices or within IPC strategies depends on the class from which it was derived, as well as on the biomaterial from which the medical device was made [2,71,97]; for example, glycolipids, lipopeptides, and Lactobacillus-derived BSs share core physicochemical properties, yet subtle differences in their surface interactions, stability, and biological effects exist. Consequently, this may affect their practical applications in real clinical settings. This comparison, therefore, evaluates them across the earlier highlighted IPC frameworks in relation to some medical devices.

#### 2.4.1. BS Antiadhesion Effects on Clinically Relevant Polymers (Silicone/PDMS)

Recent studies have shown that RLs can covalently bond to medical-grade PDMS to reduce the formation of device-associated biofilms. In a study by Dardouri et al. [32], RL-functionalized PDMS resulted in a 4.20 log (99.99%) reduction in *S. aureus* biofilm while simultaneously increasing surface hydrophilicity, with the contact angle decreasing from 95° to 17°. The same study also reported BS cytocompatibility toward human fibroblastic cells, indicated by low platelet activation and the absence of vascular irritation. Furthermore, RL-functionalized PDMS also showed activity against dual-species biofilms, with Dardouri et al. [32] reporting mixed-species reductions of up to approximately 1.9 log depending on the combination of microorganisms tested.

Studies have also shown that SLs applied by precoating or coincubation on medical-grade silicone can reduce the adhesion and biofilm formation of *S. aureus*, *P. aeruginosa*, and *C. albicans*. In a study by Ceresa et al. [32], precoating of silicone discs resulted in an approximately 75% reduction in *S. aureus* biofilms, whereas the inhibition of *C. albicans* attachment ranged from approximately 45–70% depending on the exposure time. Additionally, co-incubation assays reduced *S. aureus* and *C. albicans* adhesion/biofilm formation by 90–95%.

It has also been demonstrated that the application of lipopeptides as coatings or coincubations on silicone reduced *Candida* adhesion and early biofilm formation by ~50–70%, supporting the preventive ability of BSs on medical devices [70]. In addition, Lactobacillus-derived BSs exhibit strong antiadhesion effects on plastics and silicone against *Staphylococcus* spp. and *Candida* spp., making them ideal prophylactic conditioning agents [35,73,109].

For stable antibiofilm coatings on medical devices, RLs have been covalently bonded to PDMS surfaces through amine-based surface functionalization strategies, resulting in the production of hydrophilic and cytocompatible materials with significant antibiofilm activity against device-associated microorganisms. This covalent immobilization enhances surface modification and supports the potential use of RL-functionalized PDMS for preventing biofilm formation on medical devices [32].

In another comparative study, researchers tested SLs, RLs, and lipopeptides against mixed biofilms such as *C. albicans*–*S. aureus* and *C. albicans*–*S. epidermidis* on polystyrene and medical-grade silicone; although all three BSs reduced biofilm formation, the RLs performed best. In particular, RLs reduced their biofilm biomass and metabolic activity by approximately 94–95% in soluble form and by approximately 90–93% when used as a silicone coating. Microscopy evaluation also revealed thinner and less organized biofilms on treated surfaces [110]. This is an indication that BS coatings could help to prevent polymicrobial colonization on medical devices. However, they require further validation under realistic clinical cleaning protocols and shear conditions to confirm their translational potential [111,112].

#### 2.4.2. Disruption of Early/Established Biofilms (Device Lumens/Wet Niches)

SLs in microfluidic and flow-channel systems weaken the EPS matrix and trigger greater detachment of *P. aeruginosa* biofilms; this disruptive effect appeared to be stronger even when direct bactericidal activity is minimal [78]. RLs inhibited biofilm formation and dispersed both Gram-negative and Gram-positive bacteria in some models. Their activity is concentration-dependent and can be enhanced through chemical modifications [31,49]. Lipopeptides achieve partial disruption of fungal and bacterial biofilms, with stronger evidence supporting adhesion prevention than late-stage eradication [70,110]. With respect to disruption beyond pure prevention, SLs offer the most prominent evidence of EPS weakening and detachment, while RLs and lipopeptides provide complementary effects based on biofilm composition and mode of exposure. Table 2 summarizes the potential IPC roles of different BS classes in medical device applications.

## 3. Evidence of BS Efficacy from Device–Biofilm Models

The translational use of BSs for IPC requires experimental evaluations that demonstrate or reflect how biofilms develop on medical devices and healthcare-related surfaces. This is because the efficacy of a BS depends not only on the substrate material, but also on the hydrodynamic conditions (static or flowing) and the nature of the microbial community [33,64,101]. Therefore, understanding how static, flow-based, and microfluidic models work is important. A single model rarely captures the full spectrum of clinical conditions; thus, using several complementary systems together provides the most reliable clinical translation of BSs.

### 3.1. Static Biofilm Models

Static models are considered the most widely used starting point when evaluating biofilm formation and antibiofilm strategies [113]. In such models, outcomes may be influenced by the initial inoculum conditions and inoculation methods adopted, which can subsequently affect biofilm aggregate size, spatial arrangement, and metabolic gradients [114,115,116]. Additionally, microorganisms in static models are grown on or attached onto clinically relevant material or medical device surrogates such as silicone, PDMS, polystyrene, or titanium under completely still conditions with no flow or shear [37].

Multiple independent studies have shown that RLs, SLs, and lipopeptides consistently reduce initial adhesion and early biofilm biomass, mainly by lowering surface hydrophobicity and interfering with irreversible biofilm attachment [35,36,71,72]. However, the models used in studies can sometimes provide an overly positive view of efficacy because they fail to account for shear forces, host-protein conditioning films, and continuous nutrient exchange that occur in real healthcare environments [2,37,48]; for example, statistical models can sometimes fail to account for the structural and physiological characteristics of biofilms observed during chronic infection [18,117,118].

For the results to be truly reliable, every static assay should therefore include cytotoxicity testing at realistic exposure times, screening (especially for membrane-active BSs), and protein adsorption experiments to understand how conditioning films might affect BS effectiveness [119].

### 3.2. Flow-Based Biofilm Models

Flow-based models provide a more realistic picture of how biofilms behave on medical devices such as vascular or urinary catheters, where continuous shear stress shapes biofilm architecture and increases antimicrobial tolerance [113,120]. This is because continuous fluid flow creates shear stress, a constant nutrient supply, and waste removal, which create conditions that make biofilms denser, more structured, and more resistant than on real medical devices [69]. Under these dynamic conditions, BSs consistently reduce initial attachment, slow colonization, alter biofilm architecture, and consequently increase the susceptibility of the remaining microbial cells to co-applied antimicrobial agents. Covalently attached RL coatings remain firmly bound to surfaces and prevent microbial attachment, even under fluid shear, highlighting the importance of stable immobilization methods [32,110]. In addition, flow models also reveal IPC combinations, such as the highly lower disinfectant concentrations required once BSs have weakened the EPS matrix or increased membrane permeability. For results to have real translational value, therefore, studies must clearly report the applied shear rates, characterize surface properties before and after exposure, and include repeated maintenance cycles that mirror actual clinical device handling. However, many published flow-based studies still do not report all key hydrodynamic parameters, such as flow rate, shear stress (dynes/cm^2^ or Pa), or Reynolds number, which limits comparing models directly [67,78,91,120].

### 3.3. Microfluidic Models

Microfluidic systems can provide precise control over hydrodynamics, allowing real-time observation of early adhesion, microcolony formation, and EPS structure using microscopy. This added advantage makes them powerful for observing how BSs disrupt biofilms in ways that static or simple flow models may not capture. SLs, for example, can weaken EPS in *P. aeruginosa* biofilms and trigger detachment events even when there is no noticeable bactericidal effect [78].

Similar reductions in colonization and changes in spatial distribution have also been observed on silicone and PDMS surfaces [69]. Therefore, microfluidic platforms reveal mechanistic details that cannot be captured in static or flow models. Studies using these systems should report EPS mechanical changes, concentrations normalized relative to the CMC, and congener composition to support reproducibility and regulatory evaluation.

Although microfluidic systems provide valuable mechanistic insights, the current literature is limited by incomplete reporting of hydrodynamic and matrix-related parameters [121,122].

### 3.4. Monospecies Versus Polymicrobial Biofilm Models

Device-associated biofilm infections usually involve mixed communities of bacteria and fungi living together inside a shared extracellular matrix, which means that BS effectiveness can change noticeably depending on whether they are tested against single-species or polymicrobial biofilms. Dardouri et al. [32] demonstrated that RLs reduced biofilm colonization in the presence of three species, *P. aeruginosa*, *S. aureus*, and *Candida species*. Moreover, their findings also suggested that each bacterial species in the biofilm clearly differed in sensitivity. SLs lower adhesion and weaken the biofilm structure in bacterial–fungal models, but they usually need longer exposure times to remain effective [69]. Lipopeptides, on the other hand, are especially strong against the fungi (particularly *Candida albicans*) in polymicrobial biofilms [70].

Polymicrobial models reflect real-world conditions on medical devices more accurately than monospecies models. They are individually essential for identifying which BS classes work best against specific biofilms. These findings can guide healthcare professionals in choosing what aspects of the IPC frameworks (prevention, disruption, and/or adjunct) to adopt.

### 3.5. Translational Interpretation of the Evidence of BS Efficacy in Biofilm Models

Together, static, flow-based, and microfluidic models clarify how BSs can reduce biofilm formation and enhance IPC on medical devices, highlighting their potential in clinical and hygienic applications [123]. While static models define baseline antiadhesion activity and show that BSs can reduce initial attachment by altering surface energy and disrupting the transition to irreversible adhesion, flow models assess durability under shear stress and show that a stable surface-immobilized coating is critical for effective antiadhesion and antibiofilm effectiveness [32,34,67,124]. On the other hand, microfluidic models provide microscopic observations of early colonization and show how BSs can weaken the EPS matrix, which promotes biofilm detachment even when direct killing is not observable.

As shown in Table 3, BSs perform best as preventive and adjunctive antibiofilm agents rather than as independent antimicrobial agents in coatings. Moreover, evidence from a comparative study indicates that BSs and antibiotics exhibit complementary activity against biofilms rather than completely interchangeable functions. In the same in vitro study, broad-spectrum antibiotics were reported to be approximately 500-fold more effective against planktonic bacteria but showed limited efficacy against established biofilms. In contrast, RLs reduced biofilm biomass in established single-species systems by approximately 74–88% and in complex mixed-species biofilms by approximately 69% [31].

This contrast suggests that BSs play an important role in disrupting established biofilms where conventional antibiotics are less effective. Although combination therapies were not directly evaluated, the differing activity profiles of both BSs and antibiotics provide a clear rationale for their combined use. These complementary roles of BSs support their use as adjunctive agents with traditional antimicrobials within IPC strategies, particularly where disruption of established biofilms is needed [31,123]. Other studies have further demonstrated that RLs exhibit strong performance as durable antiadhesion coatings on device-relevant polymers. SLs show clear advantages for EPS weakening and flow-dependent biofilm disruption, including synergy with detergents or antimicrobial agents [33,78]. Lipopeptides appear to be particularly effective in fungal or polymicrobial biofilms [100].

Translational interpretation must consider that the hydrodynamics, host-protein conditioning films, nature of polymicrobial biofilms, and repeated cleaning cycles encountered during experimental laboratory analysis may differ in clinical settings. Researchers and clinicians must also realistically consider that biofilms in vivo are shaped by complex host environments, including immune responses, nutrient gradients, and microbial diversity that are not fully reproduced in vitro [2,19,112,115]. The consideration and understanding of these factors will broaden the understanding of how experimental outcomes may vary depending on initial inoculum conditions, experimental design, and factors that can substantially change BS performance against biofilms compared with simplified laboratory assays.

Future studies should therefore standardize the reporting of congener composition, CMC, and surface characterization [107], among others. In addition, cytocompatibility and hemolysis testing and BS performance under a comprehensive protocol should be prioritized [52]. Consistent reporting across these parameters can be expected to improve reproducibility, support regulatory evaluation, and guide selection of the most suitable BS class for each medical device. This perspective also aligns with broader efforts to explore microbial defense and competition systems as sources of new antimicrobial strategies, supporting the role of BSs in integrated and adjunct infection prevention approaches [125].

**Table 3:** Translational summary of BS efficacy across relevant medical devices, materials, and biofilm models.

IPC Stage	Model	BS Class	Material	BSs (Adjunct) + Antimicrobials	Target Organism(s)	Biofilm Endpoint	Outcome	Application Mode	Translational Note	Ref
**Preventive and Adjunctive**	Static	Lip	Silicone	AC7BS + AMB; AC7BS + FLZ	*Candida albicans*	MIC (planktonic), SMIC50 (biofilm)	Synergy: reduced MIC and SMIC50 vs. drugs alone; precoating AC7BS potentiates AMB	Precoating and Cotreatment	Candidate coating/adjuvant for silicone devices vs. C. *albicans* biofilms	[98]
**Preventive**	Microfluidic + static	GL	PDMS and silicone	None	*E. coli*; *S. aureus*; *Proteus vulgaris*; *Bacillus subtilis*; *P. aeruginosa*; *P. putida*	LOC visualization; CV (catheters); SEM (PDMS)	Reduced adhesion/biofilm vs. controls across channels, PDMS, silicone	Precoating	Preventive antiadhesion on device-relevant polymers	[91]
**Disruptive**	Flow-through	GL	Microfluidic channels (material n/s)	RLs + caprylic acid; RL + SLs	*P. aeruginosa* ATCC 15442; *S. aureus* ATCC 9144; mixed	Live/Dead; oxygen consumption; disruption under shear	Strong for *S. aureus*; limited for *P. aeruginosa*; combos improve	Direct perfusion under flow	Relevant to flow-shear device lumens	[120]
**Adjunctive**	Static	GL	Polystyrene/Glass	SLs + AMB/FLZ	*C. albicans*	FICI synergy; biofilm inhibition; hyphal suppression	Synergistic inhibition and killing of preformed biofilms; hyphae suppressed	Cotreatment	Adjunct to reduce antifungal dose	[126]
**Adjunctive**	Static	GL	Microplates	AZI-loaded RHL micelles (AZI@RHL)	*S. aureus*	Biomass (CV), EPS, killing, confocal disruption	48.2% biomass ↓; 92% formation ↓; 48.2% killing; EPS ↓	Cotreatment (micelles)	Improves antibiotic penetration/efficacy; dose-sparing	[94]
**Adjunctive**	Static	GL	Polystyrene	With chlorhexidine/SLSs/tetracycline/ciprofloxacin	Oral streptococci; *Actinomyces naeslundii*; *Neisseria mucosa*	MIC shift; biofilm prevention/disruption	Synergy with antimicrobials; strong antiadhesion	Cotreatment	Potential for dental materials/oral hygiene	[68]
**Adjunctive**	Static	GL	Polystyrene/Agar	SL + tetracycline (*S. aureus*); SLs + cefaclor (*E. coli*)	*S. aureus*; *E. coli*	Viability/inhibition %; SEM membrane damage	~25% ↑ inhibition (*S. aureus*) with SL + TET; ~48% ↑ (*E. coli)* with SL + cefaclor	Cotreatment	Antibiotic potentiation (rapid)	[99]
**Adjunctive**	Static	GL	Microplates	RLs + linezolid (LNZ)	LNZ-resistant *Enterococcus faecium* (3 strains)	Checkerboard (FICI), time–kill, disc diffusion, CV, CFU, qPCR	Synergy (FICI 0.25–0.5); biofilm inhibition; ~10×–100× CFU reduction in vivo	Cotreatment	Adjuvant to restore LNZ efficacy	[127]
**Adjunctive**	Static	Lip	Polystyrene	With AMP, CFZ, CRO, CIP, PIP, TOB, TMP/SMX	*E. coli* CFT073	Log CFU reduction	>1-log extra killing in most; total eradication in some pairs	Treatment of preformed biofilm	Adjunct for CAUTI-relevant biofilms	[79]
**Adjunctive**	Static	Lip	Polystyrene	Surfactin + conventional antibiotics	MRSA (DFU isolates)	MIC/MBC changes; biofilm inhibition	Surfactin active; synergy with antibiotics; biofilm inhibition	Cotreatment	Adjunct for diabetic-foot MRSA	[94]
**Adjunctive**	Static	Lip	Polystyrene	AF4/AF5 + fluconazole	*C. glabrata*	CV; XTT; EPS; ROS (DCFDA); PI; COMSTAT/CSLM	AF4/AF5 alone reduce biomass/activity; combos significantly disrupt 24 h biofilms; EPS↓; ROS↑; PI↑	Cotreatment	NAC biofilm adjunct; needs in vivo/device testing	[100]
**Adjunctive**	Microfluidic flow	GL	Microfluidic channels (material n/s)	SLs + Detergent (SDS)	*P. aeruginosa* PAO1	Catastrophic disruption; detachment; minimal bactericidal effect for SLs alone	Synergy restores potency at ~100× lower concentrations vs. single agents	Continuous perfusion	Shear-flow disruption; validate on device materials/fluids	[78]

Note: The table shows that translational success depends on matching the BS application strategy to the pathogen-specific biofilm risks most relevant to the intended device or healthcare-related surface. Abbreviations: LOC, lab-on-a-chip; Lip, lipopeptide; AC7BS, AC7 biosurfactant; AMB, amphotericin B; FLZ, fluconazole; PDMS, polydimethylsiloxane; LOC, lab-on-a-chip; CV, crystal violet; SEM, scanning electron microscopy; RL, rhamnolipid; SLs, sophorolipids; ATCC, American Type Culture Collection; FICI, fractional inhibitory concentration index; AZI, azithromycin; RHL, rhamnolipid; AZI@RHL, azithromycin-loaded rhamnolipid micelles; EPS, extracellular polymeric substance; SLSs, sodium lauryl sulphate(s); TET, tetracycline; LNZ, linezolid; CFU, colony-forming unit; qPCR, quantitative polymerase chain reaction; CAUTI, catheter-associated urinary tract infection; AMP, ampicillin; CFZ, cefazolin; CRO, ceftriaxone; CIP, ciprofloxacin; PIP, piperacillin; TOB, tobramycin; TMP/SMX, trimethoprim/sulfamethoxazole; MRSA, methicillin-resistant *Staphylococcus aureus*; DFU, diabetic foot ulcer; MIC, minimum inhibitory concentration; MBC, minimum bactericidal concentration; XTT, 2,3-bis(2-methoxy-4-nitro-5-sulfophenyl)-2H-tetrazolium-5-carboxanilide; CSLM, confocal scanning laser microscopy; COMSTAT, computerized software (software version not specified in the source article) for biofilm structure analysis; ROS, reactive oxygen species; DCFDA, 2′,7′-dichlorofluorescin diacetate; PI, propidium iodide; NAC, non-albicans *Candida*; GL, glycolipid; RLs, rhamnolipids; SL, sophorolipid; SMIC50, 50% sessile minimum inhibitory concentration; AF4, antifungal lipopeptide 4; AF5, antifungal lipopeptide 5; PAO1, *Pseudomonas aeruginosa* PAO1 strain; CFT073, *Escherichia coli* CFT073 strain; SDS, sodium dodecyl sulphate; n/s, not specified; ↑, increase/increased; ↓, reduction/reduced.

## 4. Translational Use of BSs in IPC Tools

The evidence from healthcare-relevant biofilm models described in Section 3 demonstrates that BSs are more effective as preventive and adjunctive interventions than as stand-alone antimicrobial agents. Translating these findings into clinical practice, therefore, requires their integration into established infection prevention and control (IPC) workflows, including device-care bundles, environmental hygiene programs, and antimicrobial stewardship frameworks [37,64]. Rather than replacing conventional antimicrobials, BSs should be conceptualized as adjuncts and enhancers that complement standard IPC measures to combat device-associated biofilms. These BSs directly support antimicrobial stewardship by reducing the reliance on high doses of traditional antimicrobials.

### 4.1. Device-Focused Applications

Medical devices represent a primary target for BS-based interventions because their surfaces provide favorable substrates for microbial colonization and biofilm formation. Preventive strategies aim to modify surface properties before microbial attachment occurs, thereby reducing the likelihood of persistent infection [108].

Surface coatings incorporating glycolipids or lipopeptides have been shown to reduce microbial adhesion on clinically relevant materials such as silicone and PDMS, which are commonly used in vascular catheters, urinary catheters, airway devices, and orthopedic implants [91]. Adsorption or covalent immobilization of BSs alters surface energy and hydrophobicity, creating conditions that are less favorable for stable microbial attachment. Experimental studies indicate that such coatings delay biofilm establishment and reduce biomass accumulation during early colonization stages, suggesting their potential role as prophylactic surface modifications [36,69,110]. RL coatings have also shown promising preventive antibiofilm effects on titanium implant surfaces in vitro. Early *S. aureus* and *S. epidermidis* biofilm formation was drastically reduced without detectable cytotoxicity in the study model [108].

Low-concentration BS lock or flush solutions for catheter lumens and endoscope channels can weaken matrix integrity, facilitate detachment, and enhance the efficacy of downstream antimicrobial action. Probiotic-derived BSs from *Lactobacillus species* are particularly suitable for these applications because of their reported favorable biocompatibility profiles [71,91]. However, any disruptive strategy must ensure adequate downstream antimicrobial control to minimize the risk of dispersing viable biofilms or microbial fragments to the host’s adjacent tissues. Table 4 shows some of the bacteria commonly implicated in biofilm formation in medical devices and healthcare-related surfaces.

### 4.2. Integration of BSs into Existing IPC Bundles

Effective clinical translation requires embedding BSs within established IPC bundles, rather than deploying them in isolation [32,34,48]. Current protocols already combine aseptic insertion techniques, scheduled maintenance, antimicrobial stewardship, and environmental cleaning [32,34,128]. BS coatings or adjunct solutions can be added to central-line and urinary catheters to limit initial colonization and thereby reduce the frequency or intensity of subsequent antimicrobial interventions [32,34,128]. Likewise, low-concentration BS additives can be incorporated into environmental hygiene and infection prevention strategies to slow biofilm reformation during routine disinfection cycles [48,78]. This multilayered strategy reinforces the core IPC principles of prevention and continuous risk reduction.

### 4.3. BSs and Antimicrobial Stewardship

Many BSs act against biofilms mainly through physicochemical surface modulation and EPS destabilization rather than direct bactericidal effects [36,71,78]. They may exert lower selective pressure for resistance than conventional antimicrobial coatings, although this requires dedicated comparative studies [57]. When BSs are combined with conventional antimicrobial agents, they enable substantial dose reductions while preserving or enhancing overall efficacy, directly supporting antimicrobial stewardship goals [57,78,92,127]. Therefore, the synergistic interactions documented for the biofilm models in Section 2.3.5 offer a practical route to minimize biofilm exposure to antimicrobials without reducing the overall efficacy of antimicrobial agents [57,78,94,100,127].

### 4.4. BSs: Safety, Biocompatibility, and Regulatory Considerations

Studies have shown that some BSs display low cytotoxicity and suitable compatibility with device materials at concentrations used for coatings or rinses, with probiotic-derived variants exhibiting promising biocompatibility profiles [28,36,67,129]. For instance, covalently immobilized RLs on PDMS demonstrate consistent cytocompatibility, minimal platelet activation, and no notable vascular irritation in vitro models, which is supported by detailed surface analyses that verify robust grafting stability [32,67]. Additionally, SLs maintain low toxicity on material surfaces, but more data on durability and prolonged exposure under real-world conditions are needed to drive clinical translation [28,112]. Lipopeptides, particularly, are biocompatible at typical coating doses, although careful concentration control is advised to prevent likely cytotoxic effects [95,119]. Production challenges, such as those associated with RLs sourced from *P. aeruginosa*, pose regulatory hurdles. However, researchers now focus on alternative microbial producers and refined purification methods to overcome these challenges [104,130].

Noteworthy differences among BS classes indicate that they are not suited as all-encompassing substitutes for conventional antimicrobial coatings. Instead, they offer complementary functions within infection prevention frameworks: RL–PDMS covalent systems hold strong potential for sustained antiadhesion, SLs for adjunctive disruption, lipopeptides for targeted fungal prevention, and *Lactobacillus*-derived options for biocompatible prophylaxis, albeit with needs for greater standardization [32,34,70,71,110]. Furthermore, batch variability in congener profiles, possible leaching from immobilized forms, and extended impacts from repeated clinical maintenance or host-fluid interactions necessitate a thorough assessment [104,112,131]. Regulatory hurdles and processes will vary on the basis of the application (e.g., as device coatings or maintenance aids), which will require uniform manufacturing protocols, purity criteria, and stability documentation to facilitate approval and clinical integration [104,112,132]. Thus, advancing these agents toward translational use demands a multifaceted approach, balancing their mechanistic advantages with rigorous safety and efficacy validation.

### 4.5. Practical Barriers to Clinical Adoption

Most of the evidence supporting the effectiveness of BSs comes from in vitro and other preclinical biofilm models (see Table 3) [31,103,110,126]. Because in vivo device studies and clinical trials remain limited, routine clinical adoption may be difficult without multidisciplinary approaches [103,112]. Heterogeneity in experimental conditions, BS formulations, production methods, and the cost of purified congeners may further hinder their translation into practice [104,111,112]. Additional operational barriers include compatibility with existing cleaning agents and device materials, performance under mechanical shear, integration into staff workflows, and durability across repeated device-use and maintenance cycles [48,103].

### 4.6. BS Clinical Implementation Framework

A staged, risk-based implementation strategy aligned with existing device governance and infection prevention workflows may facilitate safe clinical translation [48,112,132]. Early phases should focus on low-risk adjunctive applications, such as surface pretreatments or cleaning additives, so that compatibility and safety can be confirmed without altering core clinical procedures [48,112]. Later phases may incorporate validated coatings or maintenance protocols into device-care bundles, with monitoring of biofilm burden, device longevity, and IPC outcomes [112,128,133]. Multidisciplinary collaboration among microbiologists, IPC teams, materials scientists, clinicians, and regulatory stakeholders is essential to ensure durability, reproducibility, and compliance with safety standards [11,103,112].

## 5. Limitations, Gaps, and Research Priorities

This section builds on the mechanistic synthesis (Section 2), experimental evidence (Section 3), and translational framework (Section 4). The following section critically evaluates the current limitations and research priorities.

### 5.1. Experimental and Methodological Limitations

As previously stated, much of the current evidence on the efficacy of BSs against biofilms comes from in vitro studies which, although valuable for mechanistic insight, do not fully represent the conditions encountered in clinical settings [103,112,134]. As discussed in Section 3, static biofilm assays may overestimate efficacy because they lack shear stress, nutrient flow, conditioning films, and the biological complexity present on medical devices in vivo [18,103,117]. Flow-based and microfluidic systems offer greater physiological relevance, but these models remain highly variable across studies [103,113,120]. Limited comparability also arises because shear rates, flow regimes, material preparation, inoculum conditions, inoculation methods, and exposure times differ widely across in vitro biofilm models [111,115,116]. In addition, early experimental conditions may influence biofilm development. Differences in inoculum concentration can alter biofilm structure, oxygen gradients, and antimicrobial tolerance. This means that the outcomes observed in vitro may reflect experimental design rather than intrinsic antimicrobial or BS efficacy [115,116]. Furthermore, in vivo biofilms may exist as surface-attached communities on devices or as suspended aggregates within tissues, both of which exhibit high tolerance to antimicrobial treatment and host immune responses [19].

Variation in BS formulations, including degree of purification, congener composition, and concentration, further complicates interpretation [104,111]. BS crude extracts are frequently tested, but these can differ substantially from purified, formulated, or immobilized preparations intended for translational use [103,104].

The quantification of biofilm inhibition or disruption is also inconsistent. Some studies rely mainly on crystal violet (CV) staining, whereas others use microscopy, viable counts, metabolic assays, or EPS-related measurements, making direct comparison difficult [31,103,111]. Few studies assess structural or mechanical changes in the matrix in a standardized way, despite the central role of EPS weakening in many proposed BS mechanisms [103,104]. Most published work also focuses on short exposure periods, which creates uncertainty about long-term BSs’ sustained efficacy during repeated exposure, prolonged device use, or clinically relevant maintenance cycles [103,104].

### 5.2. BS Translational and Clinical Gaps

BS translational gaps remain obvious across static, flow-based, and microfluidic models, despite encouraging results [103,112]. Clinical trials and robust in vivo device models evaluating BS coatings, rinses, or flushes remain limited, making it difficult to judge how well current laboratory findings will translate into routine practice [31,103,134]. The long-term stability of BS layers under shear stress, repeated cleaning or maintenance cycles, and exposure to host fluids remains insufficiently understood, even though these factors are critical for catheter and other indwelling device applications. The behavior and activity of BSs in the presence of conditioning films, organic load, and host-derived factors also require further exploration. This is mainly because these variables can substantially influence adhesion, biofilm architecture, and treatment response [18,103,117].

Regulatory and manufacturing barriers present additional obstacles, including the absence of dedicated regulatory frameworks and the need for standardized analytical methods to support BS approval and clinical translation [132]. Despite these barriers, the global BS market is expanding steadily, reflecting increasing industrial and biomedical interest driven by the demand for sustainable and biocompatible surfactants, although cost and scalability remain key constraints for clinical adoption [135]. Scale-up, purification, batch-to-batch consistency, and congener variability remain unresolved challenges, particularly for BSs derived from mixed microbial products or complex fermentation systems [104]. These issues affect reproducibility, cost, and the generation of the stability and quality control data needed for regulatory evaluation [104,112]. When critically examined, the translational uncertainties of these BSs limit their immediate clinical deployment and highlight the need for standardized and device-specific evaluation pathways that combine realistic biofilm models with durability, safety, and implementation data [103,112].

### 5.3. Possibilities of Microbial Resistance to BSs

As detailed in Section 4.4, some BSs exhibit favorable safety profiles, especially those derived from Lactobacillus species [28,36,71], although long-term exposure has not been fully evaluated. The published literature specifically addressing stable microbial resistance to BSs are limited [136]. Most available studies focus instead on antimicrobial mechanisms, antibiofilm activity, membrane or surface-related effects, and translational potential rather than on repeated-exposure resistance outcomes [29,67,107,111,112].

Potential concerns include leaching from coatings, host tissue interactions, and unintended effects on the beneficial microbiota [2,32,67,131]. High concentrations of membrane-active glycolipids may also have hemolytic or cytotoxic effects, which underscores the importance of dose optimization and careful exposure design, especially for internal device coatings or maintenance solutions that may have direct contact with human tissues [28,119].

Unlike conventional antibiotics, many BSs act mainly through physicochemical surface effects, antiadhesion, membrane perturbation, and biofilm destabilization rather than through a single classical intracellular target [36,71,78]. This may reduce the selective pressure for stable AMR compared with conventional antimicrobial agents, but direct comparative evidence remains limited and this assumption should be tested explicitly [112,137]. Repeated exposure or subinhibitory concentrations could still promote adaptive responses, altered community structure, or changes in resistance gene prevalence, and these possibilities should therefore be monitored in future BS evaluations [136,138]. Future studies should include long-term adaptation studies, surveillance for reduced susceptibility, adaptive biofilm restructuring, and resistance-associated genetic markers during prolonged or repeated exposure.

### 5.4. Need for BS Standardization

Standardization is essential for advancing BS research and supporting regulatory assessment [104,112,132]. Future studies should consider harmonized model systems, consistent exposure regimens, and clearly defined performance metrics such that their findings can be compared and reproduced more reliably across laboratories [103,112]. IPC-relevant testing conditions, including polymicrobial biofilms, physiologically relevant shear, and conditioning films, should be incorporated more routinely because these factors strongly influence microbial adhesion, biofilm architecture, and treatment response [103,115,138]. Reporting standards should also include the BS’s purity, congener profile, CMC, formulation details, and surface characterization before and after treatment [104,117]. Such standardization would improve reproducibility, strengthen evidence synthesis, and increase confidence in the translational potential of BS-based interventions [103,112].

### 5.5. Future Research Priorities for BSs

Future research on BSs should focus on closing the methodological and translational gaps identified above, as well as introducing artificial intelligence and metabolic engineering. The integration of artificial intelligence into BS research may offer promising opportunities to model complex biological systems and optimize fermentation processes. This may lead to the production of BSs with enhanced antimicrobial and antibiofilm properties, thereby accelerating translational development [112,139,140,141]. Priority areas include long-term evaluation of BS coatings under clinically relevant hydrodynamic conditions, testing in polymicrobial and fungal-biofilm models, and safety assessment in biological systems that truly reflect host tissues and microbiota [18,67,103,132]. Device-specific studies of BSs, particularly for catheters and other associated materials, will be important for determining whether promising laboratory findings can translate into meaningful reductions in biofilm burden and infection risk [32,34,108].

Pilot implementation studies examining BS integration into device-care bundles, lock or flush strategies, or environmental cleaning workflows are also needed to assess feasibility in practice. These studies should include outcomes such as biofilm burden, durability of intervention, device longevity, workflow compatibility, and infection-related endpoints [67,103,112,117]. Cross-disciplinary collaboration among microbiologists, materials scientists, clinicians, IPC specialists, and regulatory stakeholders is essential to support responsible translation and ensure that BS-based interventions meet the expectations for safety, durability, reproducibility, and practical applications [11,112,132].

## 6. Conclusions

BSs are a class of biomolecules that address persistent biofilm-related challenges in healthcare. This review demonstrates that glycolipids, lipopeptides, and probiotic-derived surfactants consistently reduce early microbial adhesion, weaken extracellular matrices, and enhance antimicrobial penetration into early/established biofilms. These effects portray the preventive, disruptive, and adjunctive roles of BSs within infection prevention and control frameworks, especially when BSs are deployed on clinically relevant materials such as silicone and PDMS and evaluated under hydrodynamic conditions that reflect real device environments.

Most mechanistic and experimental evidence supports BSs primarily as surface-modifying, EPS-weakening, and synergistic agents rather than as substitutes for conventional antimicrobial agents. Their greatest potential lies in preventive surface conditioning, disruptive maintenance strategies, and antimicrobial stewardship through dose reduction and improved penetration. The class-specific strengths of BSs further refine their applications: RLs are promising as durable antiadhesion coatings, SLs are effective disruptive agents under flow, and lipopeptides provide added benefit in combating fungal or mixed biofilms.

Limited long-term data, the lack of clinical trials, and variability in formulations and testing protocols currently restrict BS translation in healthcare and remain significant challenges. Standardization of BS characterization, reproducibility under shear, safety evaluation, and congener profiling will be essential to progress toward their regulatory approval and clinical adoption. Future work on their translations should prioritize realistic device-specific polymicrobial models, safety assessments under repeated exposure, and pilot studies that integrate BSs into device-care bundles and environmental hygiene workflows in healthcare settings.

Additionally, the integration of artificial intelligence with microbiological and biochemical research may play a key role in advancing BS-based strategies for infection prevention and control.

If these gaps can be addressed, BS-based strategies have the potential to become valuable adjuncts within IPC frameworks. This will support safer medical devices, reduce reliance on high-dose antimicrobial agents, and contribute to broader efforts to mitigate AMR, thus compromising modern medicine. By shifting IPC toward preventive surface engineering and early biofilm control, BSs may help to shape a more sustainable and resilient approach to infection prevention in modern healthcare.

## Figures and Tables

**Table 1:** Conceptual framework of preventive, disruptive, and adjunctive BS application strategies for device-associated IPC.

Strategy Type	Device Type	Mode of Application	Primary IPC Objective	Expected Mechanism		Key Advantages	Risks Limitations	Safeguards/Paired Controls	Ideal IPC Use-Case	Translational Readiness
**Preventive**	Catheters, implants	Surface coating; pretreatment, polymer incorporation	Prevent adhesion	Surface energy modulation	Compliance-independent; reduces downstream antimicrobial demand	Loss of activity over time; fouling; cleaning compatibility		Durability and compatibility testing	Routine prophylaxis for high-risk devices	Conceptual/preclinical
**Disruptive**	Catheter lumens; reusable medical device	Lock solution; flush; decontamination	Destabilize early or established biofilms	EPS destabilization; matrix hydration; increased antimicrobial penetration	Adjunctive control/removal	Biofilm seeding; inconsistent exposure; cleaning compatibility		Protocolized pairing with antimicrobials or disinfectant exposure; defined dwell times; seeding monitoring	Device salvage or targeted decontamination of lumens	Conceptual/preclinical
**Adjunctive**	Infected devices; high-risk surfaces	Dual agent (BSs + antimicrobials)	Synergistic clearance; overcome resistance	Enhances antimicrobial access; cell membrane permeabilization	Reduces antimicrobial burden; enhances cleaning efficacy; stewardship support	Dual-agent dosing complexity; potential dual-agent incompatibility		Detailed pharmacokinetics/pharmacodynamics modelling	Complex infections	Preclinical; research stage

The framework summarizes three applications/functions of BSs as agents against microbial/biofilm attachments in IPC. Preventive applications focus on antiadhesion and surface conditioning before irreversible microbial attachment. Disruptive applications target early or established biofilms through EPS weakening and detachment. Adjunctive BSs enhance the efficacy of antibiotics, disinfectants, or cleaning agents through increased permeability and, consequently, antimicrobial penetration. The adjunctive stage enhances the reduction in standard antimicrobial agents, thereby supporting antimicrobial stewardship. These mechanisms also support reduced colonization, delayed biofilm formation, and enhanced biofilm removal during maintenance procedures.

**Table 2:** Comparison of major BS classes for IPC-relevant device applications.

BS Class	Primary IPC Role	Strongest Evidence	Key Limitation	Translational Readiness
Rhamnolipids	Preventive antiadhesion coatings	PDMS covalent immobilization studies	Manufacturing and regulatory considerations	High (coating strategies)
Sophorolipids	Disruption and antimicrobial synergy	Flow-based and microfluidic biofilm models	Durability under clinical cleaning conditions unclear	Medium–High
Lipopeptides (e.g., surfactin)	Antifungal prevention and early biofilm inhibition	Silicone *Candida* biofilm studies	Cytotoxicity thresholds at higher concentrations	Medium
Lactobacillus-derived BSs	Preventive surface conditioning and antiadhesion	Antiadhesion studies on plastics and silicone	Standardization and formulation variability	Emerging

Overall, the comparison shows that different BS classes are better suited to different IPC strategies in medical devices.

**Table 4:** Priority biofilm-forming pathogens and their implications for BS use in medical devices and environmental IPC.

Priority Pathogen or Group	Common IPC Context	Biofilm Features Relevant to IPC	Translational Implications for BSs
*S. aureus* (and related *staphylococci*)	Indwelling catheters; implants; wounds	Rapid adhesion; persistence on device materials	Strong rationale for preventive antiadhesion surfaces and adjunctive synergy
*P. aeruginosa*	Wet reservoirs; device-associated infections	Robust EPS; high tolerance; flow-dependent biofilms	Prioritize flow-based models and environmental hygiene applications
*Enterococcus species*	High-risk care settings, outbreaks, devices, and surfaces	Stress tolerance; polymicrobial participation	Test in mixed-species models and workflow-compatible interventions
*Candida species*	Catheters; silicone or polymer devices; mixed biofilms	Intrinsic tolerance; fungal–bacterial synergy	Include fungal and mixed consortia, not bacterial monospecies only

Table 4 summarizes key microorganisms associated with device-related and environmental healthcare biofilms, highlighting biofilm characteristics and implications for preventive and adjunctive BS strategies within IPC workflows.

## Data Availability

No new data were created or analyzed in this study. Data sharing is not applicable to this article.

## Abbreviations

The following abbreviations are used in this manuscript:

AC7BS	AC7 biosurfactant
AF4	Antifungal lipopeptide 4
AF5	Antifungal lipopeptide 5
AI-2	Autoinducer-2
AMB	Amphotericin B
AMP	Ampicillin
AMR	Antimicrobial resistance
ATCC	American Type Culture Collection
AZI	Azithromycin
AZI@RHL	Azithromycin-loaded rhamnolipid micelles
BS	Biosurfactant
BSs	Biosurfactants
CAUTI	Catheter-associated urinary tract infection
CAUTIs	Catheter-associated urinary tract infections
CFBS	Cell-free biosurfactant
CFU	Colony-forming unit
CFZ	Cefazolin
CIP	Ciprofloxacin
CLABSI	Central line-associated bloodstream infection
CLABSIs	Central line-associated bloodstream infections
CMC	Critical micelle concentration
COMSTAT	Computerized software for biofilm structure analysis
CRO	Ceftriaxone
CSLM	Confocal scanning laser microscopy
CV	Crystal violet
DCFDA	2′,7′-dichlorofluorescin diacetate
DFU	Diabetic foot ulcer
DOI	Digital object identifier
eDNA	Extracellular DNA
EPS	Extracellular polymeric substance
FICI	Fractional inhibitory concentration index
FLZ	Fluconazole
GL	Glycolipid
HCAIs	Healthcare-associated infections
HMW	High-molecular-weight
IPC	Infection prevention and control
Lip	Lipopeptide
LMW	Low-molecular-weight
LNZ	Linezolid
LOC	Lab-on-a-chip
MBC	Minimum bactericidal concentration
MDAIs	Medical device-associated infections
MIC	Minimum inhibitory concentration
MRSA	Methicillin-resistant *Staphylococcus aureus*
MRS	De Man, Rogosa and Sharpe
NAC	Non-albicans *Candida*
n/s	Not specified
PDMS	Polydimethylsiloxane
PI	Propidium iodide
PIP	Piperacillin
qPCR	Quantitative polymerase chain reaction
QS	Quorum sensing
RL	Rhamnolipid
RLs	Rhamnolipids
ROS	Reactive oxygen species
SDS	Sodium dodecyl sulphate
SEM	Scanning electron microscopy
SF/SFs	Surfactin/Surfactins
SL	Sophorolipid
SLs	Sophorolipids
SLSs	Sodium lauryl sulphate(s)
SMIC	Sessile minimum inhibitory concentration
SMIC50	50% sessile minimum inhibitory concentration
TEM	Transmission electron microscopy
TET	Tetracycline
TOB	Tobramycin
TMP/SMX	Trimethoprim/sulfamethoxazole
v/v	Volume/volume
WHO	World Health Organization
XTT	2,3-bis(2-methoxy-4-nitro-5-sulfophenyl)-2H-tetrazolium-5-carboxanilide
↑	Increase/increased
↓	Reduce/reduction
